# RET/PTC rearrangement in papillary thyroid carcinoma arising in malignant struma ovarii with abdominal wall metastasis and cervical thyroid gland: a case report and review of the literature

**DOI:** 10.1186/s13044-023-00181-5

**Published:** 2023-09-27

**Authors:** Maryam Kabootari, Reza Habibi Tirtashi, Azita Zadeh-Vakili, Maryam Zarkesh, Hossein Samadanifard, Shirin Haghighi, Fereidoun Azizi, Atieh Amouzegar

**Affiliations:** 1 Metabolic Disorders Research Center, Golestan University of Medical Sciences, Gorgan, Iran; 2 Prevention of Metabolic Disorders Research Center, Research Institute for Endocrine Sciences, Shahid Beheshti University of Medical Sciences, Tehran, Iran; 3 Endocrine Research Center, Research Institute for Endocrine Sciences, Shahid Beheshti University of Medical Sciences, No. 24, Aerabi St, Daneshjoo Blv, Velenjak, P.O. Box 19395-4763, Tehran, Tehran Iran; 4 Cellular and Molecular Endocrine Research Center, Research Institute for Endocrine Sciences, Shahid Beheshti University of Medical Sciences, Tehran, Iran; 5 Department of Endocrinology, School of Medicine, Hazrat-e Rasool Hospital, Iran University of Medical Sciences, Tehran, Iran; 6 Department of Hematology and Medical Oncology, Shahid Beheshti University of Medical Sciences, Tehran, Iran

**Keywords:** Case Reports, Struma ovarii, Papillary thyroid carcinoma, Molecular Pathology, ret-PTC-1 oncoprotein

## Abstract

**Background:**

Struma ovarii refers to rare mature cystic teratomas containing at least 50% of thyroid tissue, and malignant transformation is known to be even rarer. The synchronous development of malignant struma ovarii and cervical thyroid carcinoma are also scarce and poorly understood due to limited data about molecular features. Here, we present the first report of RET/PTC 1 rearrangement in synchronous metastatic malignant struma ovarii to the abdominal wall and cervical thyroid cancer.

**Case presentation:**

We described a 47-year-old multigravida woman with bilateral adnexal and lower abdominal wall masses detected during the evaluation of abnormal uterine bleeding. The patient underwent a hysterectomy, bilateral salpingo-oophorectomy, and surgical removal of abdominal wall mass. Then, the pathological evaluation revealed papillary thyroid carcinoma (PTC) within struma ovarii and metastatic PTC in the abdominal wall fibro adipose tissue. Further, cervical thyroid gland physical examination and ultrasound illustrated a nodule within the left lobe. Subsequently, a total thyroidectomy was performed, and a histological examination revealed PTC. Furthermore, all affected tissue, i.e., struma ovarii, abdominal wall metastasis, and cervical thyroid gland tested for BRAF and RAS mutations and RET/PTC 1 rearrangement. RET/PTC 1 rearrangement was identified among all three different sites. Finally, after six years of follow-up, the patient had no evidence of recurrence or distant metastasis.

**Conclusions:**

In light of these findings, malignant struma ovarii might yield a clue to cervical thyroid carcinoma, and the molecular analysis could provide valuable information for understanding the underlying mechanism, tumor clinicopathological behaviors, and prognosis.

## Background

Mature cystic teratomas (dermoid cysts), the most common ovarian germ cell tumor type, constitute 20% of all ovarian neoplasms [1]. Thyroid tissue could be found in approximately 20% of mature cystic teratomas, and 5% of thyroid-containing teratomas are classified as struma ovarii, composed entirely or predominantly (≥ 50%) of thyroid tissue. Most cases are benign and malignant transformation might occur in 5–10% of patients. Papillary thyroid carcinoma (PTC) is the most common malignancy in struma ovarii. According to previous investigations, metastases are found in 5–25% of patients suffering from malignant struma ovarii. The metastases from malignant struma ovarii are primarily found in ascites or peritoneal washing fluid and pelvic peritoneum or structures [2–6].

Previous reports described synchronous development of malignant struma ovarii and primary thyroid carcinoma, which occurred in 5–10% of patients [2, 3, 6–9]. Molecular analysis was conducted among a few previous reports of the malignant struma ovarii and coexistent cervical thyroid carcinoma, including BRAF(V600E), N-RAS, H-RAS, KIT, TERT-promotor mutations, and RET-PTC rearrangements [2, 7, 8, 10–14]. Some previous investigations claimed synchronous cervical thyroid cancer or genetic abnormalities like BRAF and RAS mutations were associated with higher risk and more aggressive disease [15, 16]. However, due to data insufficiency, the impact of the mutation on patients’ prognosis and survival was not fully understood [9]. Considering the overall rarity of malignant struma ovarii and the probability of synchronous cervical thyroid cancer, more investigations are needed to reveal the essential role of molecular alterations.

To our best knowledge, only one case report described the coexistence of cervical PTC and metastatic malignant struma ovarii alongside molecular analysis [2]. Thus, we present another case of metastatic malignant struma ovarii and coexistent cervical PTC, along with molecular analysis, after six years of follow-up, accompanied by a similar literature review.

## Case presentation

A 47-year-old woman, multigravida, was referred to evaluate her abnormal uterine bleeding that started two years ago. Previous ultrasound showed multiple intramural subserosal uterine myomas (15–20 mm diameter) and a simple cyst (20 mm diameter) in the right ovary without septation and solid component.

**Fig. 1:**
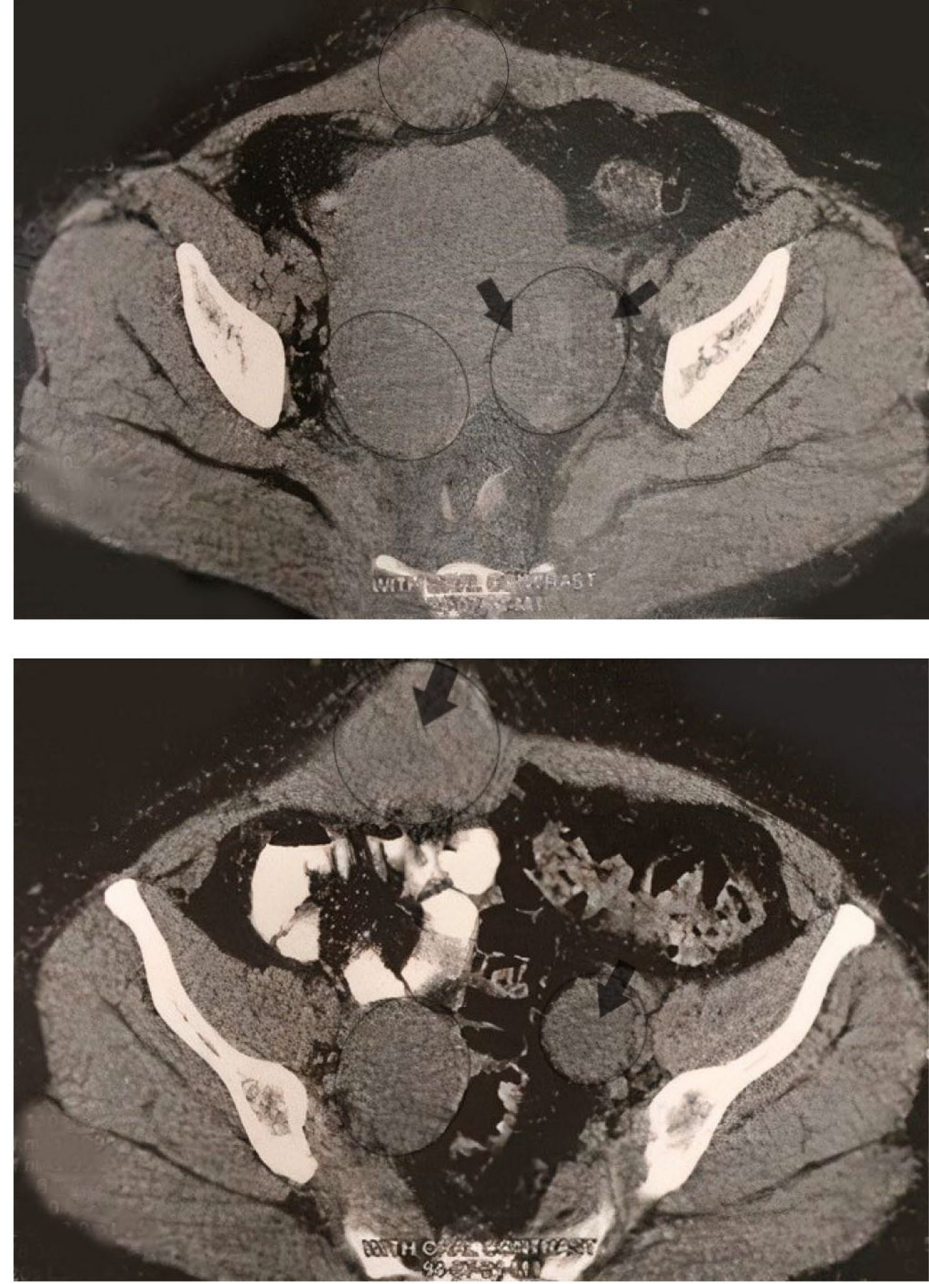
Bilateral smooth marginated solid-cystic masses in the pelvic cavity within ovarian sites, alongside an isodense mass lesion with a prominent solid component in the right rectus muscle in the lower abdomen

The patient’s symptoms had worsened over seven months, and she also suffered from a sense of abdominal fullness. The abdominal physical examination was unremarkable except small palpable mobile mass located in the lower abdomen. Recent abdominal and transvaginal ultrasounds illustrated bilateral adnexal masses (51 × 31 mm diameter simple cyst in the right ovary and 38 × 29 mm diameter hyperechoic mass in the left ovary). Furthermore, there was a new finding; a hypoechoic mass located deep in the soft tissue of the lower abdominal wall (63 mm in diameter). Moreover, computerized tomography (CT) of the abdomen and pelvis confirmed an abdominal mass within the left ovary and a mass in the abdominal wall. (Fig. 1)

**Fig. 2:**
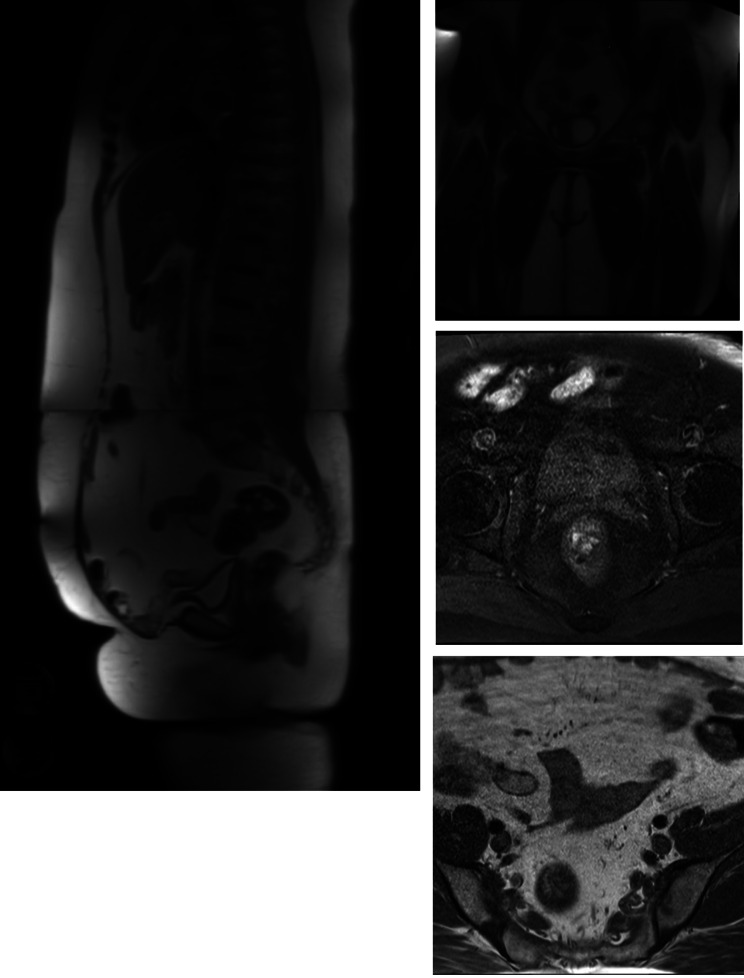
Post-surgical abdominal Magnetic Resonance Imaging. T1 Images in the sagittal plane, coronal plane, and axial plane of fat saturation and non-fat saturation

Considering all findings, in November 2015, the patient underwent a total abdominal hysterectomy, bilateral salpingo-oophorectomy, and surgical removal of the abdominal wall mass. Both ovaries were removed intact, and peritoneal washing was performed. The pathological evaluation described a 28 mm focus of PTC within struma ovarii without angiolymphatic invasion surrounded by normal thyroid tissue, confined to the ovary. Moreover, evaluation of abdominal wall mass revealed a metastatic PTC in fibro adipose tissue. In addition, there was no evidence of thyroid-type tumor cells in peritoneal washing fluid, and all surgical margins were free of tumor cells. Post-surgical Magnetic Resonance Imaging results are available in Fig. 2.

**Fig. 3:**
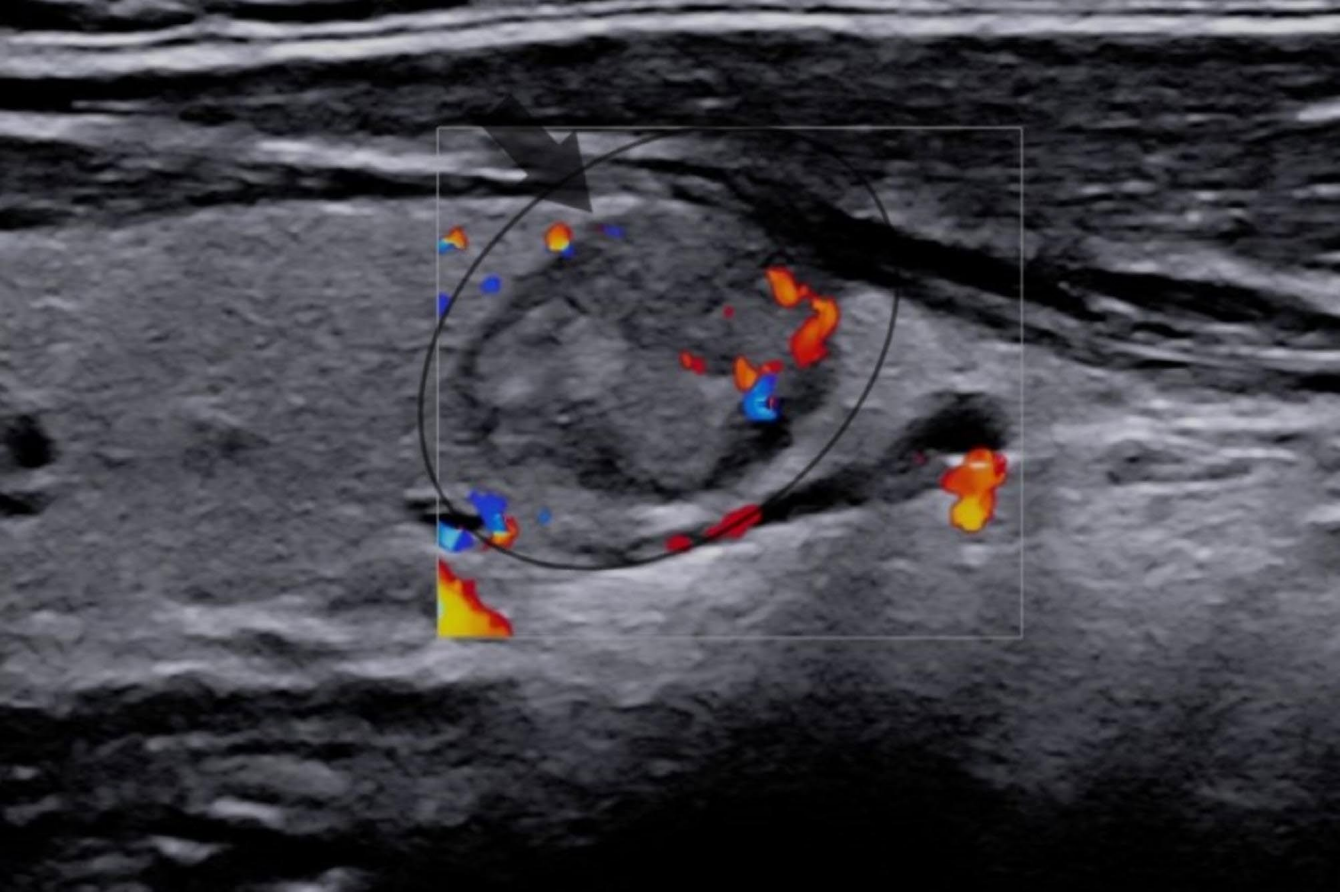
A 21 mm solid nodule within the left lobe of the cervical thyroid gland with hypoechogenicity, lobulated borders, vertical direction, and macrocalcification. (TI-RADS: 5) TIRADS, Thyroid imaging reporting and data system.

The patient did not complain about thyroid dysfunction symptoms and denied radiation exposure and previous cervical thyroid carcinoma in herself or her family. The examination of the cervical thyroid gland and lymph nodes was unremarkable, except for a nodule within the left lobe. Then, thyroid ultrasound showed a 21 mm nodule within the left lobe (Fig. 3) without cervical lymph nodes suspecting malignancy, and subsequent ultrasound-guided fine-needle aspiration biopsy illustrated PTC. Furthermore, laboratory evaluations revealed that the complete blood count, biochemical parameters, and thyroid function tests were normal. In light of these findings, the patient underwent a total thyroidectomy, and the pathological evaluation demonstrated bilateral and multifocal PTC with no evidence of angiolymphatic invasion, extrathyroidal extension, or cervical lymph node metastasis. [Stage 1 (T1N0M0)-ATA low risk] The left lobe evaluation showed a malignant nodule (15 mm diameter) with a mixed papillary and follicular variant of PTC. Moreover, four foci of papillary thyroid microcarcinoma (5 mm diameter) were found in the right lobe.

We examined the pathogenic variants of BRAF and N-RAS genes and RET/PTC1 rearrangement. Samples were collected from affected tissues, including malignant struma ovarii, cervical thyroid gland, and abdominal wall metastasis.

Three to four tissue curls cut (10 μm) from Formalin-Fixed Paraffin-Embedded (FFPE) blocks were obtained from Tehran Khatam-al-Anbya Hospital. Total DNA and RNA were extracted using TRIzol reagent (Invitrogen U.S. Cat. No. 15596-026) according to the manufacturer’s instructions. Codon 600 mutation in BRAF oncogene and codon 13 mutation in N-RAS were investigated by direct sequencing. Exon 15 of the BRAF gene and Exon number 2 from N-RAS were amplified by polymerase chain reaction (PCR) using previously reported primers [17]. After quality control by running 2% agarose gel electrophoresis, direct DNA sequencing was carried out using the same primers by Applied Biosystems 3130/3130xl Genetic Analyzers. Total RNA (1 μg) was reverse transcribed with a cDNA synthesis kit (Thermo Fisher Scientific, USA) according to the manufacturer’s protocol to determine the RET/PTC1 rearrangement [18]. The amplification condition was optimized by quantitative reverse transcriptase real-time PCR (qRT-PCR) using the Rotor-Gene 6000 (Corbett Research, Sydney, Australia). The results are explained in Table 1.

**Table 1:** Genetic abnormalities of papillary thyroid carcinoma residing in the cervical thyroid gland, malignant struma ovarii, and abdominal wall metastasis

Tumor Site	BRAF V600E	N-RAS	RET/PTC1
Cervical thyroid gland	**-**	**-**	**+**
Malignant struma ovarii	**-**	**-**	**+**
Abdominal wall metastasis	**-**	**-**	**+**

One month afterward, the patient received oral administration of 100 mCi ^131^I for treatment, and one week later, she underwent a whole-body scintigraphy. The scan showed radiotracer uptake in the remnant of the thyroid tissue in the thyroid bed. Furthermore, the absence of abnormal tracer activity throughout the body illustrated no evidence of regional or distant metastasis, and the patient did not receive post-surgical radiotherapy or chemotherapy.

**Fig. 4:**
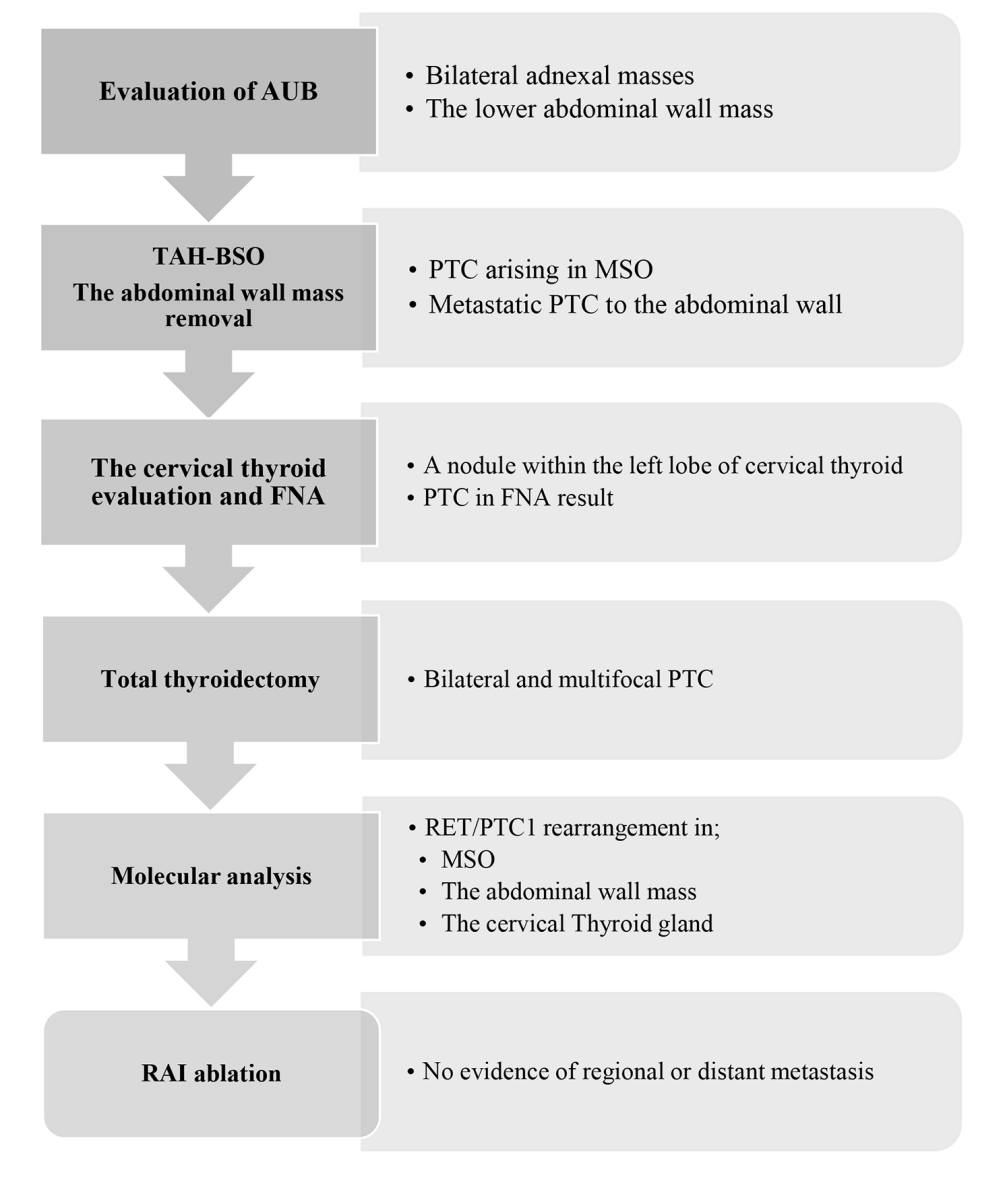
The patient’s medical history step by step AUB, abnormal uterine bleeding; TAH-BSO, total abdominal hysterectomy- bilateral salpingo-oophorectomy; PTC, papillary thyroid carcinoma; MSO, malignant struma ovarii; FNA, fine-needle aspiration biopsy; RAI, radioactive iodine

The patient’s whole medical history is summarized step by step in Fig. 4.

After six years of follow-up, the patient had no recurrence or distant metastasis complaint. Moreover, neck ultrasound and CT scan of abdomen and pelvis have revealed nothing related to recurrence or metastasis, alongside undetectable thyroglobulin and thyroglobulin antibody levels.

## Discussion and conclusions

The current report describes a unique synchronous metastatic malignant struma ovarii and cervical PTC after six years of follow-up, alongside molecular analysis that revealed positive RET/PTC1 rearrangement among three different sites, i.e., malignant struma ovarii, cervical thyroid gland, and abdominal wall metastasis.

The coexistence of malignant struma ovarii and cervical thyroid carcinoma is quite rare. Among previous reports, we found eighteen publications that described synchronous malignant struma ovarii and cervical thyroid carcinoma. The characteristics of these reports are shown in Table 2, and treatment and follow-up data are also available in Table 3.

**Table 2:** Coexistence of malignant struma ovarii and cervical thyroid carcinoma case reports characteristics

Number	Reported case	Age(years)	Presentation	MSO histopathology	Metastasis	CTC histopathology	Genetic profiling
1	Lim et al. [19] 2008	63	Palpable mass in the neck	FVPTC	No evidence	PTC Lymph node metastases	Unknown
2	Janszen et al. [28] 2008	52	Unexplained weight loss	FTC	No evidence	Papillary microcarcinoma	Unknown
3	Marti et al. [7] 2012	44	Palpable adnexal mass	PTC	No evidence	PTC ETE + Central lymph node metastasis	BRAF(V600E) -
4	Leite et al. [29] 2013	78	Pelvic pain Significant weight loss The large mass in RLQ	PTC LV invasion -	No evidence	FVPTC LV invasion – ETE -	Unknown
5	Leong et al. [2] 2013	42	Growing left pelvic mass	PTC LV invasion-	Positive peritoneal washing	Multifocal PTC ETE + FVPTC Perithyroidal lymph node metastasis	BRAF(V600E) - K-RAS - RET-PTC -
6	Krishnamurthy et al. [30] 2013	51	Abdominal pain and distension Abdominopelvic mass	FVPTC	No evidence	FVPTC	Unknown
7	Brusca et al. [31] 2015	30	Abdominal pain Increased steady weight The palpable large mass in RLQ and hypogastric region	FVPTC	No evidence	Papillary microcarcinoma	Unknown
8	Aguilera et al. [32] 2015	57	Right ovarian mass	PTC	No evidence	Papillary microcarcinoma	Unknown
9	Ma et al. [10] 2016	47	Sudden onset RLQ pain Emesis Vaginal spotting	PTC FVPTC Tall cell variant of PTC	No evidence	Papillary microcarcinoma FVPTC	KIT mutation +
10	Capitao et al. [13] 2017	35	Ovarian mass in a routine pelvic exam	FVPTC	No evidence	WDT-UMP ETE – LV invasion -	BRAF(V600E) -
11	Middlebeek et al. [11] 2017	55	Incidental thyroid nodule	PTC	No evidence	FVPTC ETE - LV invasion -	BRAF(V600E) -
12	Boyd et al. [33] 2017	30	Acute RLQ pain	PTC LV invasion -	No evidence	PTC ETE -	Unknown
13	Gomes-Lima et al. [8] 2018	62	Accidentally Pelvic mass in CT scan	PTC	No evidence	PTC LV invasion - ETE -	HRAS Q61R + in PTC within thyroid gland NRAS Q61R + in PTC within struma ovarii
14	Tzelepis et al. [12] 2019	32	unknown	FVPTC LV invasion +	No evidence	PTC ETE +	BRAF(V600E)- TERT promoter mutations-
15	Seigel et al. [3] 2019	55	Weight loss Intermittent fatigue	PTC	No evidence	PTC	Unknown
16	Gonzalez-Cejudo et al. [34] 2021	57	Incidental CT scan finding	PTC	No evidence	Papillary microcarcinoma	Unknown
17	Li et al. [9] 2021	44	Accidental finding of pelvic mass	PTC	No evidence	PTC ETE -	Unknown
18	Donato et al. [14] 2021	35	Accidental finding of pelvic mass	FVPTC LV invasion -	No evidence	FVPTC LV invasion - ETE – Bilateral lung metastases (micronodules)	BRAF(V600E)-

Abbreviations: MSO, malignant struma ovarii; CTC, cervical thyroid carcinoma, FVPTC, follicular variant papillary thyroid carcinoma; PTC, papillary thyroid carcinoma; FTC, follicular thyroid carcinoma; ETE, extrathyroidal extension; LV, lymphatic/vascular; RLQ, right lower quadrant; WDT-UMP, well-differentiated thyroid tumor of uncertain malignant potential; CT, computerized tomography

**Table 3:** Coexistence of malignant struma ovarii and cervical thyroid carcinoma treatment and follow-up

Number	Reported case	parity	Treatment	Follow-up	Recurrence
1	Lim et al. [19] 2008	Unknown	Total thyroidectomy Radical neck dissection Unilateral salpingo-oophorectomy	Unknown	Unknown
2	Janszen et al. [28] 2008	Para 3 / postmenopausal	BSO Total thyroidectomy RAI ablation	24 months	No evidence
3	Marti et al. [7] 2012	Unknown	TAH-BSO Omentectomy Pelvic and paraaortic lymph node dissection	9 months	No evidence
4	Leite et al. [29] 2013	Unknown	Unilateral salpingo-oophorectomy Thyroid resection RAI ablation	24 months	No evidence
5	Leong et al. [2] 2013	Unknown	TAH-BSO Total thyroidectomy RAI ablation	12 months	No evidence
6	Krishnamurthy et al. [30] 2013	Multigravida	TAH-BSO Omentectomy Lymph nodes and peritoneal sampling Total thyroidectomy RAI ablation	6 months	No evidence
7	Brusca et al. [31] 2015	Unknown	Unilateral salpingo-oophorectomy Total thyroidectomy RAI ablation	6 months	No evidence
8	Aguilera et al. [32] 2015	Unknown	Oophorectomy Total thyroidectomy RAI ablation	unknown	unknown
9	Ma et al. [10] 2016	Unknown	TAH-BSO Total thyroidectomy RAI ablation	36 months	No evidence
10	Capitao et al. [13] 2017	Unknown	Unilateral oophorectomy Total thyroidectomy RAI ablation	Unknown	Unknown
11	Middlebeek et al. [11] 2017	Unknown	Total thyroidectomy RAI ablation BSO	Unknown	Unknown
12	Boyd et al. [33] 2017	Nulliparous	Unilateral salpingo-oophorectomy Total thyroidectomy RAI ablation	Unknown period	No evidence
13	Gomes-Lima et al. [8] 2018	Unknown	BSO Total thyroidectomy RAI ablation	60 months	No evidence
14	Tzelepis et al. [12] 2019	Unknown	Unilateral salpingo-oophorectomy Partial omentectomy Total thyroidectomy RAI ablation	12 months	No evidence
15	Seigel et al. [3] 2019	Para 3 / postmenopausal	Unilateral salpingo-oophorectomy Total thyroidectomy RAI ablation	Unknown	unknown
16	Gonzalez-Cejudo et al. [34] 2021	Unknown	Unilateral salpingo-oophorectomy Total thyroidectomy RAI ablation	60 months	No evidence
17	Li et al. [9] 2021	Unknown	Unilateral salpingo-oophorectomy Total thyroidectomy	6 months	No evidence
18	Donato et al. [14] 2021	Gravida 3/ para 3	Unilateral salpingo-oophorectomy Total thyroidectomy RAI ablation	51 months	No evidence

Abbreviations: BSO, bilateral salpingo-oophorectomy; RAI, radioactive iodine; TAH, total abdominal hysterectomy

The mean diagnosis age of synchronous malignant struma ovarii and cervical thyroid cancer was 48.26 years (range from 30 to 78 years), and most patients complained about abdominal/pelvic symptoms at the onset of the disease. Nevertheless, two patients [11, 19] suffered from neck problems, and the post-total thyroidectomy ^131^I scan demonstrated the struma ovarii diagnosis. According to pathological evaluation, PTC was the most common histologic subtype in malignant struma ovarii and cervical thyroid gland. Furthermore, molecular analysis was documented in eight reports [2, 7, 8, 10–14]. BRAF (V600E) mutation was the most common genetic evaluation, followed by RAS, KIT, TERT promoter mutations, and RET-PTC rearrangements. However, all evaluation results were negative except for RAS mutations in Gomes-lima et al. [8] and KIT mutation in Ma et al. [10]. Moreover, most patients underwent a similar treatment approach; unilateral or bilateral salpingo-oophorectomy, total thyroidectomy, and RAI ablation. Follow-up data were also available for twelve patients, and the average follow-up time was 23.15 months (range of 6 to 60 months) with no evidence of recurrence or distant metastasis.

The coexistence of malignant struma ovarii and cervical thyroid carcinoma represents synchronous multifocal thyroid-type tumors in distinct anatomical locations without lymphatic connection. This phenomenon may be explained by hypothesized “field cancerization” and early genomic instability. “Field cancerization” or “field effect” refers to prolonged exposure to carcinogens leading to independent genetic alterations at thyroid-type tissue in different topographical sites. On the other hand, the initial genetic instability occurs during embryogenesis, and separated affected cells differentiate into thyroid-type tissue in distinct anatomical locations. Then, lifetime parallel carcinogen exposures lead to independent synchronous tumorigenesis. In other words, genetic predispositions, environmental exposure, and epidemiological factors contribute to multiple preneoplastic lesions arising synchronously [2, 20–22].

To our best knowledge, only one report presented the coexistence of metastatic malignant struma ovarii and cervical PTC alongside molecular analysis. Leong et al. [2] described a 42-year-old woman with a growing left pelvic mass that underwent total abdominal hysterectomy and bilateral salpingo-oophorectomy. The pathological evaluation revealed papillary thyroid carcinoma arising from struma ovarii, alongside the existence of thyroid-type tumor cells in the peritoneal washing fluid. Then, the total thyroidectomy histological evaluation illustrated bilateral and multifocal PTC along with extrathyroidal extension and metastasis to the perithyroidal lymph node. Similar to Leong et al. study, our patient’s disease presented by abdominal manifestation, then she underwent the same treatment approach, and pathology evaluation revealed PTC among struma ovarii, cervical thyroid gland, and abdominal wall metastasis. Moreover, in contrast to the study of Leong et al., cervical thyroid histological evaluation demonstrated no evidence of extrathyroidal extension or lymph node metastasis, and our patient’s distant metastasis occurred in the abdominal wall, which was not a common site for malignant struma ovarii metastasis. Furthermore, to explore genetic alterations, Leong et al. evaluated BRAF(V600E) and KRAS mutations and RET/PTC 1 and 3 rearrangements, and all results were negative. Conversely, we performed BRAF(V600E), NRAS mutations, and RET/PTC 1 rearrangement among malignant struma ovarii, cervical thyroid gland, and abdominal wall metastasis, and RET/PTC 1 rearrangement was found among all affected tissues. Finally, there was no evidence of recurrence or metastasis during the one-year follow-up of the Leong et al. study and the six-year follow-up of our study.

RET/PTC is defined as intrachromosomal rearrangement of the long arm of chromosome 10. According to previous studies among PTC patients, the incidence of RET/PTC rearrangements was 2.5–67% and had a relatively high prevalence in radiation-induced PTC [23, 24]. Furthermore, RET/PTC rearrangements were associated with advanced tumor stage and a higher risk of distant metastasis [25]. However, the prognostic role of RET/PTC rearrangement in PTC still meets with controversy [23]. Reviewing previous publications, we found only two reports documented RET/PTC rearrangement in struma ovarii; first, Elisei et al. [26] reported a benign struma ovarii with RET/PTC 3 rearrangement in a 59-year-old woman. Second, Boutross-Tadroset et al. [27] described seven RET/PTC rearrangements in follicular variant of PTC within malignant struma ovarii, one patient with RET/PTC 3 and others with RET/PTC 1. To our best knowledge, we present the first RET/PTC 1 rearrangement report in synchronous metastatic malignant struma ovarii and cervical PTC in a patient without previous radiation exposure.

The strength of the current study was a quite rare case of synchronous malignant struma ovarii with abdominal wall metastasis and cervical PTC, along with detection of RET/PTC 1 rearrangement in molecular analysis and a long period of follow-up. Regardless of these strengths, our study had some limitations. First, we did not evaluate other RET/PTC rearrangements and clonal origins of the tumors. Second, immunohistochemistry results were not available.

In conclusion, considering the rarity of synchronous malignant struma ovarii and cervical thyroid carcinoma, finding malignant struma ovarii might be a clue to probable cervical thyroid cancer. Hence, examinations and imaging of the cervical thyroid gland should be considered among malignant struma ovarii patients. Moreover, the post-diagnosis molecular analysis could provide helpful information for understanding the underlying mechanism of coexisting disease, the clinicopathological behavior of the tumors, and the patient’s prognosis.

## Data Availability

All data and materials are available upon request.

## List of abbreviations

CT: Computerized tomography
PCR: Polymerase chain reaction
PTC: Papillary thyroid carcinoma

## References

[1] Peterson WF, Prevost EC, Edmunds FT, Hundley JM, Morris FK (1955). Benign cystic teratomas of the ovary: a clinico-statistical study of 1,007 cases with a review of the literature. Am J Obstet Gynecol.

[2] Leong A, Roche PJ, Paliouras M, Rochon L, Trifiro M, Tamilia M (2013). Coexistence of malignant struma ovarii and cervical papillary thyroid carcinoma. J Clin Endocrinol Metabolism.

[3] Siegel MR, Wolsky RJ, Alvarez EA, Mengesha BM (2019). Struma ovarii with atypical features and synchronous primary thyroid cancer: a case report and review of the literature. Arch Gynecol Obstet.

[4] Wei S, Baloch ZW, LiVolsi VA (2015). Pathology of struma ovarii: a report of 96 cases. Endocr Pathol.

[5] Cui Y, Yao J, Wang S, Zhao J, Dong J, Liao L (2021). The clinical and pathological characteristics of malignant struma Ovarii: an analysis of 144 published patients. Front Oncol.

[6] Ayhan S, Kilic F, Ersak B, Aytekin O, Akar S, Turkmen O (2021). Malignant struma ovarii: from case to analysis. J Obstet Gynecol Res.

[7] Marti JL, Clark VE, Harper H, Chhieng DC, Sosa JA, Roman SA (2012). Optimal surgical management of well-differentiated thyroid cancer arising in struma ovarii: a series of 4 patients and a review of 53 reported cases. Thyroid.

[8] Gomes-Lima CJ, Nikiforov YE, Lee W, Burman KD (2018). Synchronous independent papillary thyroid carcinomas in struma ovarii and the thyroid gland with different RAS mutations. J Endocr Soc.

[9] Li S, Yang T, Xiang Y, Li X, Zhang L, Deng S (2021). Clinical characteristics and survival outcomes of malignant struma ovarii confined to the ovary. BMC Cancer.

[10] Ma D, Guseva NV, Dahmoush L, Robinson RA (2016). Struma ovarii with malignant transformation and germline KIT mutation: a case report with review of the literature. Int J Gynecol Pathol.

[11] Middelbeek RJ, O’Neill BT, Nishino M, Pallotta JA (2017). Concurrent intrathyroidal thyroid cancer and thyroid cancer in struma ovarii: a case report and literature review. J Endocr Soc.

[12] Tzelepis EG, Barengolts E, Garzon S, Shulan J, Eisenberg Y. Unusual case of malignant struma ovarii and cervical thyroid cancer preceded by ovarian teratoma: case report and review of the literature. Case Reports in Endocrinology. 2019;2019. 10.1155/2019/7964126 PMC6441504 31007958

[13] Capitao R, Saraiva C, Santos F, Ferrinho C, Roque C, Bello C, et al. editors. Malignant struma ovarii and synchronous tumour of thyroid gland in the same patient: a single pathway for two different tumours? Endocrine Abstracts; 2017.

[14] Donato S, Simões H, Leite V (2021). Malignant struma ovarii with concurrent thyroid Cancer: outcomes during and after pregnancy. Eur Thyroid J.

[15] Addley S, Mihai R, Alazzam M, Dhar S (2021). Malignant struma ovarii: surgical, histopathological and survival outcomes for thyroid-type carcinoma of struma ovarii with recommendations for standardising multi-modal management. A retrospective case series sharing the experience of a single institution over 10 years. Arch Gynecol Obstet.

[16] Chung SY, Chi J, Park J, John V, Seetharamu N (2021). Malignant struma ovarii with late recurrence harbouring high microsatellite instability. BMJ Case Reports CP.

[17] Zarkesh M, Zadeh-Vakili A, Azizi F, Fanaei SA, Foroughi F, Hedayati M. The association of BRAF V600E mutation with tissue inhibitor of metalloproteinase-3 expression and clinicopathological features in papillary thyroid cancer. Int J Endocrinol Metabolism. 2018;16(2). 10.5812/ijem.56120 PMC5972213 29868127

[18] Puxeddu E, Moretti S, Giannico A, Martinelli M, Marino C, Avenia N (2003). Ret/PTC activation does not influence clinical and pathological features of adult papillary thyroid carcinomas. Eur J Endocrinol.

[19] Lim ST, Jeong H-J, Chung M-J, Yim C-Y, Sohn M-H (2008). Malignant struma ovarii demonstrated on post-therapy radioiodine scan after total thyroidectomy for papillary thyroid cancer. Clin Nucl Med.

[20] Jones TD, Wang M, Eble JN, MacLennan GT, Lopez-Beltran A, Zhang S (2005). Molecular evidence supporting field effect in urothelial carcinogenesis. Clin Cancer Res.

[21] Lu Z, Sheng J, Zhang Y, Deng J, Li Y, Lu A (2016). Clonality analysis of multifocal papillary thyroid carcinoma by using genetic profiles. J Pathol.

[22] Su X, Chen S, He K, Mao Z, Ruan J, Zhou J (2019). Clonal analysis of early-stage bilateral papillary thyroid cancer identifies field cancerization. Endocrine.

[23] Romei C, Ciampi R, Elisei R (2016). A comprehensive overview of the role of the RET proto-oncogene in thyroid carcinoma. Nat Reviews Endocrinol.

[24] Henderson YC, Shellenberger TD, Williams MD, El-Naggar AK, Fredrick MJ, Cieply KM (2009). High rate of BRAF and RET/PTC dual mutations associated with recurrent papillary thyroid carcinoma. Clin Cancer Res.

[25] Vuong HG, Altibi AM, Duong UN, Ngo HT, Pham TQ, Tran HM (2017). Role of molecular markers to predict distant metastasis in papillary thyroid carcinoma: promising value of TERT promoter mutations and insignificant role of BRAF mutations—a meta-analysis. Tumor Biology.

[26] Elisei R, Romei C, Castagna MG, Lisi S, Vivaldi A, Faviana P (2005). RET/PTC3 rearrangement and thyroid differentiation gene analysis in a struma ovarii fortuitously revealed by elevated serum thyroglobulin concentration. Thyroid.

[27] Boutross-Tadross O, Saleh R, Asa SL (2007). Follicular variant papillary thyroid carcinoma arising in struma ovarii. Endocr Pathol.

[28] Janszen EW, Van Doorn HC, Ewing PC, De Krijger RR, De Wilt JH, Kam BL (2008). Malignant struma ovarii: good response after thyroidectomy and 131i ablation therapy. Clin Med Oncol.

[29] Leite I, Cunha TM, Figueiredo JP, Félix A (2013). Papillary carcinoma arising in struma ovarii versus ovarian metastasis from primary thyroid carcinoma: a case report and review of the literature. J Radiol case Rep.

[30] Krishnamurthy A, Ramshankar V, Vaidyalingam V, Majhi U (2013). Synchronous papillary carcinoma thyroid with malignant struma ovarii: a management dilemma. Indian J Nuclear Medicine: IJNM: Official J Soc Nuclear Med India.

[31] Brusca N, Del Duca SC, Salvatori R, D’Agostini A, Cannas P, Santaguida MG et al. A case report of thyroid carcinoma confined to ovary and concurrently occult in the thyroid: is conservative treatment always advised? Int J Endocrinol Metabolism. 2015;13(1). 10.5812/ijem.18220 PMC4338667 25745492

[32] Aguilera BG, Vázquez RG, Herguido NG, Gallego FS, González EN (2015). The lack of consensus in management of malignant struma ovarii. Gynecol Endocrinol.

[33] Boyd JC, Williams BA, Rigby MH, Kieser K, Offman S, Shirsat H (2017). Malignant struma ovarii in a 30-year old nulliparous patient. Thyroid Res.

[34] González-Cejudo C, Calderón AM, García-Arreza A, Vieites B, Martínez M (2021). Conservative surgical approach of a synchronous malignant ovarian struma and papillary thyroid carcinoma in a postmenopausal woman. J Obstet Gynaecology: J Inst Obstet Gynecol.

